# Supplementary material for: Impact persistence of stock market risks in commodity markets: Evidence from China
Source: PLoS One. 2021 Nov 8;16(11):e0259308. doi: 10.1371/journal.pone.0259308 (PMC8575302; doi:10.1371/journal.pone.0259308)
Supplement: Attachment — Submitted filename: Report PONE-D-21-17518.pdf [file pone.0259308.s001.pdf]

Ref: *PONE-D-21-17518*

Title: *Impact Persistence of Stock Market Risks on Commodity Market Risks*

Journal: *PLOS ONE*

This paper analyses the impact persistence of volatility spillover and illiquidity spillover from the stock market to commodity markets for the case of China. This manuscript addresses an interesting question. However some revisions should be performed in order to elevate the overall quality of the paper. I present my suggestions and comments below.

1. I would suggest to revise the title, emphasizing the geography (China) of your paper.

2. I would suggest to substantially rewrite the Abstract in order to clearly emphasize what exactly is the main contribution of this paper.

Please be more concrete and assertive while writing the Abstract. For example, how the first two sentences of the Abstract (see below) help the reader to comprehend what is the paper about?:

<<*The risk spillover among financial markets has been investigated in a burgeoning number of studies. Two types of risks are emphasized: the risk of market return fluctuation, namely, volatility, and the risk of market liquidity fluctuation, namely, illiquidity.*>>

This statement generates more questions than answers. For example, why are just two types of risks (volatility and liquidity) emphasized in the literature? What is about the credit risk, reputation risk, operational risk, etc.?

Then you continue: <<*Against this backdrop, we scrutinize ...*>>. What exactly is this backdrop you are talking about? In my view, in the first two sentences of the Abstract you have not managed to duly explain this point.

It is not clear what do you mean under the 10 periods, during which the stock market volatility shock persists in the corn and soybean markets.

I would suggest indicating the time frame(s) of your empirical study in the Abstract.

3. The literature survey must be considerably improved in order to position the paper among the related state of the art. I suggest to include a separate section providing a

thorough literature review, focusing on the most recent studies. Authors may consider to address the following papers among many others:

Ahmed, A.D., Huo, R. (2021), Volatility transmissions across international oil market, commodity futures and stock markets: Empirical evidence from China. *Energy Economics*, 93, 104741, https://doi.org/10.1016/j.eneco.2020.104741.

Frömmel, M., Han, X., Li, Y., Vigne, S. (2021), Low liquidity beta anomaly in China. *Emerging Markets Review*, 100832, https://doi.org/10.1016/j.ememar.2021.100832.

Umar, Z., Gubareva, M., and Teplova, T. (2021), The impact of Covid-19 on commodity markets volatility: Analyzing time-frequency relations between commodity prices and coronavirus panic levels. *Resources Policy*, 73, 102164. https://doi.org/10.1016/j.resourpol.2021.102164

Umar, Z., Gubareva, M., Naeem, M., and Akhter, A. (2021), Return and volatility transmission between oil price shocks and agricultural commodities. *PLOS ONE*. 16(2): e0246886. https://doi.org/10.1371/journal.pone.0246886.

Zhang, Y., Ding, S. (2021), Liquidity effects on price and return co-movements in commodity futures markets. *International Review of Financial Analysis*, 76, 101796, https://doi.org/10.1016/j.irfa.2021.101796.

4. While describing on page 2 (lines 40-45) the main distinguishing features of your research, you abruptly continue with the following text:

<<Furthermore, our study is also comparable with the risk spillover research in advanced economies, such as the stock illiquidity spillover in G7 stock markets (see Andrikopoulos et al., 2014).>>

Please, use the “bridge” phrase to prepare the reader for changing of the direction of your thought from presenting distinguishing features of your research to describing the similarities of your study with the already published research papers.

Moreover, I suggest you to explain in what extent your paper is comparable to the paper by Andrikopoulos et al., 2014, and other more recent papers on this topic.

5. Please explain what is the advantage of the VAR model in the context of your research. Why don’t you employ other alternatives, such as Markov switching model and/ or multiplicative error model (MEM), among others?

6. Please explain what is the advantage of the Amihud measure of liquidity? Why don’t you utilize other alternatives? Authors may address the following recent studies on liquidity measures, while addressing this point:

Díaz, A. and Escribano, A. (2020), Measuring the multi-faceted dimension of liquidity in financial markets: A literature review. *Research in International Business and Finance*, 51, 101079, https://doi.org/10.1016/j.ribaf.2019.101079.

Cakici, N. and Zaremba, A. (2021), Liquidity and the cross-section of international stock returns. *Journal of Banking & Finance*, 127, 106123, https://doi.org/10.1016/j.jbankfin.2021.106123.

Gubareva, M. (2021), Covid-19 and high yield emerging market bonds: insights for liquidity risk management. Risk Management. https://doi.org/10.1057/s41283-021-00074-7

Kang, W. and Zhang, H. (2014), Measuring liquidity in emerging markets, Pacific-Basin Finance Journal, 27, pp 49-71, https://doi.org/10.1016/j.pacfin.2014.02.001.

7. Are the results of your paper are robust? I suggest to address the robustness of the results.

8. While the results are quite interesting, there is no thorough discussion of the results. Authors should provide economic interpretation of the obtained results and explain what are the implications of this study, how this study could be used by practitioners, by policy-makers etc.. I suggest to introduce the additional section to address this point.

Summarizing, I found the subject of this research to be interesting. However, Authors need to elevate the quality of this paper to be eligible for publishing in *PLoS One* in accordance with the suggestions above.

I thank the Authors for the opportunity to consider this manuscript.

Sincerely,

Reviewer
